# Predicting Lymphoma Development by Exploiting Genetic Variants and Clinical Findings in a Machine Learning-Based Methodology With Ensemble Classifiers in a Cohort of Sjögren's Syndrome Patients

**DOI:** 10.1109/OJEMB.2020.2965191

**Published:** 2020-02-14

**Authors:** Konstantina D. Kourou, Vasileios C. Pezoulas, Eleni I. Georga, Themis Exarchos, Costas Papaloukas, Michalis Voulgarelis, Andreas Goules, Andrianos Nezos, Athanasios G. Tzioufas, Ηaralampos M. Moutsopoulos, Clio Mavragani, Dimitrios I. Fotiadis

**Affiliations:** ^1^ Unit of Medical Technology and Intelligent Information Systems, Department of Materials Science and EngineeringThe University of Ioannina 37796 GR45110 Ioannina Greece; ^2^ Department of Biological Applications and TechnologyThe University of Ioannina 37796 GR45110 Ioannina Greece; ^3^ Department of InformaticsIonian University 68997 GR49100 Corfu Greece; ^4^ Foundation for Research and Technology-HellasInstitute of Molecular Biology and BiotechnologyDepartment of Biomedical Research 54578 Ioannina GR45110 Greece; ^5^ Department of Pathophysiology, School of MedicineNational and Kapodistrian University of Athens 68993 GR15772 Athens Greece; ^6^ Department of Physiology, School of MedicineNational and Kapodistrian University of Athens 68993 GR15772 Athens Greece; ^7^ Academy of Athens 69100 GR10679 Athens Greece

**Keywords:** Ensemble methods, genetic variants, lymphoma prediction, machine learning, Sjögren's Syndrome

## Abstract

Lymphoma development constitutes one of the most serious clinico-pathological manifestations of patients with Sjögren's Syndrome (SS). Over the last decades the risk for lymphomagenesis in SS patients has been studied aiming to identify novel biomarkers and risk factors predicting lymphoma development in this patient population. *Objective:* The current study aims to explore whether genetic susceptibility profiles of SS patients along with known clinical, serological and histological risk factors enhance the accuracy of predicting lymphoma development in this patient population. *Methods:* The potential predicting role of both genetic variants, clinical and laboratory risk factors were investigated through a Machine Learning-based (ML) framework which encapsulates ensemble classifiers. *Results*: Ensemble methods empower the classification accuracy with approaches which are sensitive to minor perturbations in the training phase. The evaluation of the proposed methodology based on a 10-fold stratified cross validation procedure yielded considerable results in terms of balanced accuracy (GB: 0.7780 ± 0.1514, RF Gini: 0.7626 ± 0.1787, RF Entropy: 0.7590 ± 0.1837). *Conclusions:* The initial clinical, serological, histological and genetic findings at an early diagnosis have been exploited in an attempt to establish predictive tools in clinical practice and further enhance our understanding towards lymphoma development in SS.

## I. Introduction

Sjögren's syndrome (SS) is a chronic autoimmune disorder mainly manifested with dryness of mucosae as a result of exocrine gland involvement chiefly the salivary and lachrymal glands resulting in dry eyes and mouth. Systemic features also occur, in one third of the patients, as a result of skin, lungs, kidneys, liver and vessel involvement. Lymphoma development is one of the most serious manifestations. [1], [2].

Over the last decades a large amount of data revealed several clinical (salivary gland enlargement, purpura, Raynaud [3], [4]), hematological, serological (RF, Ro/La autoantibodies, monoclonal gammopathy [4]–[6], low complement C4 [3], serum BAFF [7], [8], sFLT [9]) and histopathological features (extensive lymphocytic infiltration [10]), as predictors for lymphoma development in Sjögren's syndrome. Of interest, these risk factors usually present at disease onset implying that a distinct genetic background could characterize the subgroup of SS patients which will develop lymphoma in the course of their disease [11].

On this basis, genetic variants of genes implicated in the regulation of chronic inflammation such as TNFAIP3 [12]–[14] and LILRA3 [15], B cell activation [16], [17], type I IFN pathways such as TREX-1 [18] as well as epigenetic processes [19] have been shown to increase the risk of Non-Hodgkin Lymphoma (NHL) in SS. The susceptibility to lymphoma development increases especially in patients in whom the disease starts before the age of 40 years old, as evidenced by the higher frequencies of the BAFF-R [17], [20], TNFAIP3 [12] and LILRA3 [15] variants.

Lymphoma prediction based on clinical and biological predictors have been studied in terms of statistical analysis and prediction rules [4], [21]–[24]. Towards this direction, in [4] a predictive tool in clinical practice has been developed for SS-related lymphoma development based on the initial clinical, laboratory and histopathological variables of SS patients Data mining algorithms have been also exploited for the identification of patient subgroups and the prediction of lymphoma in primary SS [25]. The associations among patient's demographics, clinical and serological variables have been defined and a prediction model based on Artificial Neural Networks (ANNs) has been developed able to predict new unseen records with high sensitivity and specificity. In the present study, we aim to identify the contribution of combined initial clinical, serological and histopathological features with genetic variants in predicting lymphoma development using Machine Learning-based (ML) methodology with ensemble classifiers. We focused on the development of a ML-based methodology able to classify accurately new patients according not only to their traditional clinical findings but also to their genetic susceptibility as a critical factor that predispose to lymphoma development in SS patients. The proposed methodology is based on the Gradient boosting (GB) [26] and Random Forest (RF) [27] ensemble classifiers for developing the predictive models which are characterized by the ability to generalize their decision boundaries to regions where there are no available training examples. This type of classifiers was selected in terms of the variance and bias estimation which contribute to the expected error of a classification model. The novelty of the proposed ML-based methodology pertaining to the potential usefulness of genetics in predicting lymphoma development in SS patients. The classification results reported in our study are obtained from stratified 10-fold cross validation with the ensemble classifiers outperforming the single Logistic Regression (LR) approach and the Support Vector Machine (SVM) classifier. Based on our results, we anticipate that the current work could provide new insights into the aggressive behavior of lymphoma development in SS patients.

## II. Results

**Figure 1.**
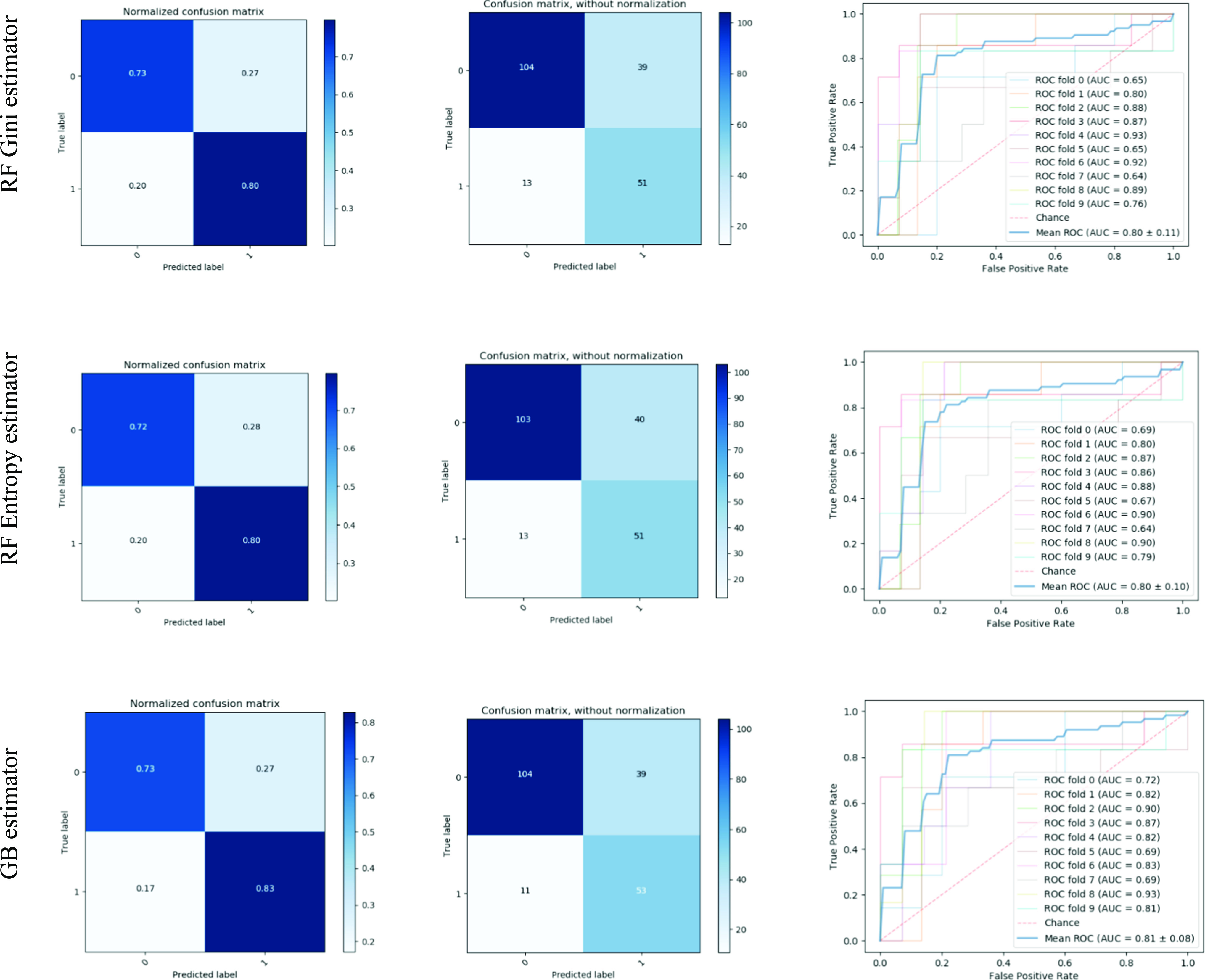
The normalized and non-normalized confusion matrices obtained for each classification model. The ROC curves after the evaluation of models’ performance are also illustrated. Each row corresponds to the respective classifier's evaluated performance. In the upper side the classification performance of RF Gini estimator is depicted (confusion matrices and ROC curve). In the middle and lower side of the figure the classification results of RF Entropy and GB classifiers are presented, respectively. The ROC curves correspond to the mean ROC curves and auc after applying the 10-fold cross validation procedure in the proposed ML methodology. The ROC curve in each fold is also illustrated for comparison purposes. In addition, the ± 1SD is also given with the mean ROC.

Fig. 1 presents the evaluation performance of the GB and RF ensemble classifiers. More details are provided within the supplementary material regarding the obtained results, the study cohort, the preprocessing steps and the model training and parameter tuning of the ensemble classifiers. For the RF classifier both Gini and entropy criterion were applied in order to determine the best way to split the samples. These measures are defined according to the fraction of samples that belong to class $i$ at a given node $t$. The best split is then selected according to the degree of impurity of the child nodes [32]. Three input cases were considered in the current study for comparison reasons and for assessing the models’ performances (Table III supplementary material). More specifically, the clinical phenotype of each patient along with the genetic data were considered (input case 1) for building the proposed predictive models and further evaluate their performance. For assessing the potential of combining the initial SS patient's medical features with genetic variants in predicting lymphoma development, we followed the same procedure for input case 2 (the clinical phenotype for each patient) and input case 3 (the genotyped data acquired for each patient) and evaluated the models’ performances in terms of certain metrics and hyper-parameter optimization criterion (i.e., balanced accuracy). For each prediction model (i.e., RF models and GB model) the mean value of each metric is presented along with the computed standard deviation (Table III supplementary material).

We can observe that the combination of the initial clinical, serological and histopathological features with genetic variants result in the accurate prediction of lymphoma development in SS patients with considerable high balanced accuracy for RF Gini (0.7626 ± 0.1787), RF Entropy (0.7590 ± 0.1837) and GB (0.7780 ± 0.1514) classifiers, respectively (Table III supplementary material). We should also report for input case 1 (clinical and genetic data), the remarkable results obtained with reference to the sensitivity metric implying the high proportion of patients with lymphoma who have been predicted as positive by the classifiers (RF Gini classifier: 0.8000 ± 0.3435, RF Entropy classifier: 0.8000 ± 0.3435 and GB classifier: 0.8309 ± 0.2594) (Table III supplementary material and Fig. 1). As illustrated in the confusion matrices (Fig. 1), the GB model could predict more subjects as true positives (104) and true negatives (53) in comparison to RF Gini and RF models. The mean AUC of the models in terms of the sensitivity and specificity results are 0.7988 ± 0.2186 (RF Gini classifier), 0.7995 ± 0.1917 (RF Entropy classifier) and 0.8054 ± 0.1570 (GB classifier) which constitute promising results for predicting lymphoma development (Fig. 1).

For input case 2 (clinical data) the GB classifier performed better with slightly higher mean AUC (0.8215 ± 0.1534) in comparison to the mean AUC of input case 1 (clinical and genetic data) (0.8054 ± 0.1570). The exploitation of only the clinical patient records could be comparable with the combination of both genotyped data and the clinical phenotypes towards predicting lymphoma development. However, we can observe that the computed sensitivity, positive predictive and negative predictive values of the GB model for input case 2 (clinical data) are lower enough in accordance to the respective evaluation metrics for input case 1 (clinical and genetic data). Concerning the exploitation of individual genetic variants for building the predictive models (input case 3) the results yielded by the proposed methodology are moderate with significantly lower balanced accuracy, sensitivity and specificity in comparison to input case 1 and input case 2. Based on this knowledge, we can admit that the combination of both data sources (clinical and genetic profile) could result in more accurate classification results by obtaining predictive models with reference to ML techniques.

**Figure 2.**
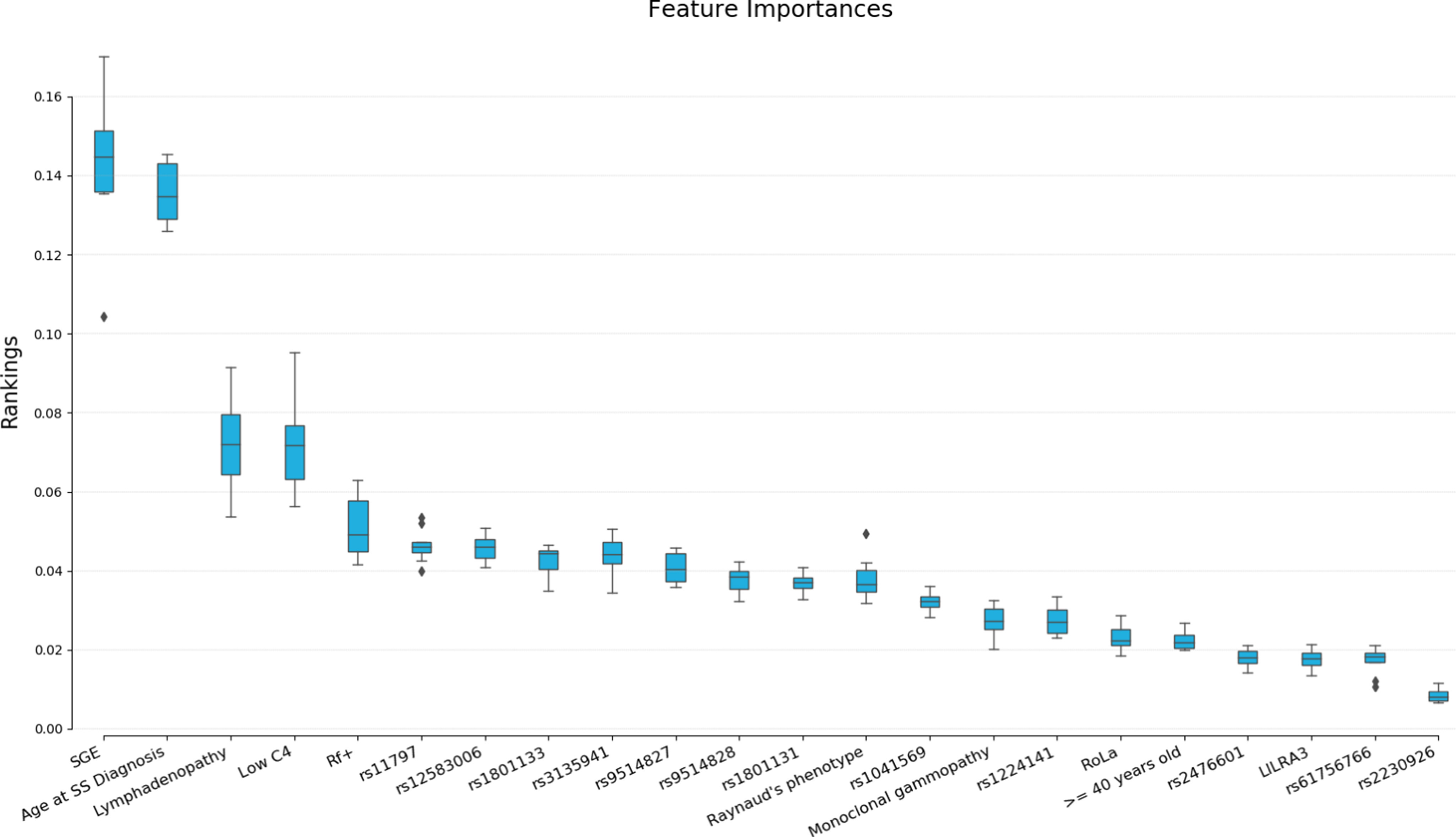
Boxplot with the mean feature rankings for each variable considered by the respective estimator. RF feature selection was performed with threshold the “mean” and “max_features” equal to the max number of features in the dataset considered at each experiment (input case 1clinical and genetic data).

Fig. 2 illustrates the boxplot with mean feature importances according to the feature selection and ranking procedure performed with RF selector (section V. MATERIALS AND METHODS). Hence, the most important features which contribute to accurate and unbiased predictions of lymphoma development were identified. We can observe that the 10 most informative features are SGE, age at SS diagnosis, low C4, lymphadenopathy, RF plus, BAFF, TREX and MTHFR677 SNPs. We can observe that beyond these features, the most informative ones are mainly clinical findings and the rs11797 and rs12583006 reference numbers of the corresponding genetic variants. We shall recall that the presented values refer to the mean importance rankings.

## III. Discussion

Predicting the risk for lymphoma development still remains a clinical unmet need in SS. The main clinical and genetic aspects of this major complication need to be elucidated for providing a meaningful clinical impact and translational findings in the field.

In this study, we highlight the potential of combining the clinical, serological and histological parameters along with the genetic profile of SS patients for the prediction of lymphoma development through a ML methodology consisting of ensemble algorithms. GB and RF classifiers were utilized to obtain accurate classification results based on their generalization ability and the minimization of errors in the training phase [33]–[36]. Based on the selected estimators in the inner ensemble, the training phase was conducted on different balanced bootstrap samples while random under-sampling was considered [34], [35]. Feature ranking was applied in terms of the RF selector based on importance weights. The threshold value used for feature selection and ranking was set to the maximum number of variables within our dataset. The number of features ranked by the estimator was 22, with SGE and age at SS diagnosis being the most important features that contribute to the classification of patients’ samples (mean ranking of SGE = 0.1446, mean ranking of age at SS diagnosis = 0.1347). rs12583006 and rs11797 genetic variants are also included within the first 10 most informative features contributing to the prediction of lymphoma development (mean ranking of rs12583006 = 0.0462, mean ranking of rs11797 = 0.0460). The feature ranking results (Fig. 2) confirmed the identification of SGE and lymphadenopathy as independent adverse predictors for NHL development. We should also note that the age of patients at disease diagnosis could be a potential predictor for lymphoma development. According to published results, mucosa-associated lymphoid tissue (MALT) lymphoma occurs in younger pSS patients [37] which indicates the severity of diagnosis at an early stage. Furthermore, the rs11797 and rs12583006 genetic variants have been found as significant predictors along with specific clinical findings.

**Figure 3.**
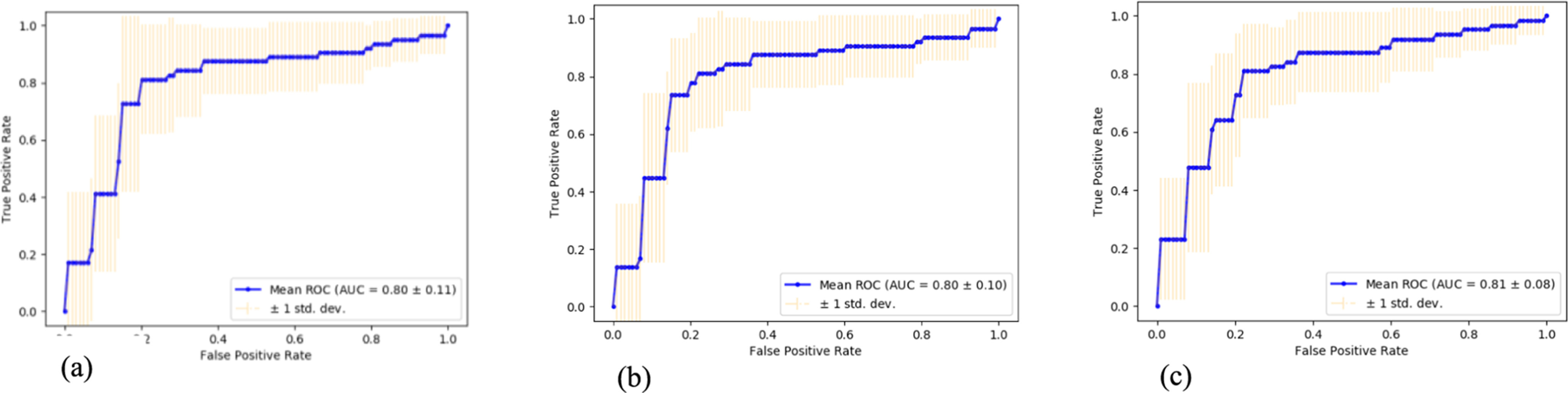
The calculated mean ROC curve and auc (a–c), with the variance of each curve when the training set is split into 10 different subsets. This pinpoints how the estimator output is affected by changes in the training data, and how different the splits are from one another in 10-fold cross validation. The left ROC curve corresponds to RF Gini estimator and the middle and right ones to RG Entropy and GB classifiers, respectively.

The mean ROC curves of RF Gini, RF Entropy and GB predictive models, with reference to input case 1 (clinical and genetic data), are also depicted in Fig. 3(a-c), including the variance of each curve based on the different subsets created when the training sets are splitted. The figures exhibit how the classifiers output is affected by changes in the training data and how different the subsets are from one another according to the cross-validation procedure. We can observe the low variance which is closely related to the robustness of our methodology. We can also observe the remarkably high results achieved by the three classifiers in terms of the negative predictive value metric (Table III supplementary material). This constitutes a promising impact of our methodology in predicting accurately the patients that are found as negatives and actually do not have diagnosed with lymphoma during SS progression. As illustrated in Fig. 1, the high sensitivity values were obtained when both initial findings and genetic variants are exploited. This reveals the ability of the developed classification models to predict with high proportion the patients who have lymphoma and are truly predicted as positive. To evaluate the predictions on the test sets, different scores were also applied besides the balanced accuracy criterion, such as the f1 score, the log loss metric and the recall. However, the results obtained were similar or with very slight differences in comparison to the balanced accuracy scoring parameter. The proposed methodology was also applied to different input cases where the clinical and genetic variants were considered separately (Table III supplementary material). Obviously, the exploitation of the genotyped data from the patients result in moderate classification balanced accuracy related to the risk for lymphoma development.

On the contrary, individual clinical, serological and histopathological parameters have been identified in the literature as major predictors of B cell lymphomas. This is in accordance with the reported ML-based classification results (input case 2 in Table III supplementary material) revealing the superiority of collecting both the initial parameters and the genetic data on the disease onset. In the present work, we highlight the need for identifying risk clinical phenotypes in combination with the patients’ genetic profiles for predicting the development of lymphomas which constitutes a major complication of SS. We show that the integration of both the patient's genetic background and the clinical phenotype could enhance the prediction accuracy of our ML models while improving disease diagnosis (Table III supplementary material). We further validated the methodology with other supervised learning methods used for classification, such as SVM (with linear kernel) and LR [4], [8]. Given the reported results based on the exploitation of both data types, we demonstrated that the proposed methodology with the ensemble classifiers outperforms the model performance based on SVM and LR. The reported balanced accuracy and AUC for SVM are 0.6395 ± 0.2540 and 0.6934 ± 0.2586, respectively. The evaluated performance for the LR predictive model resulted in balanced accuracy 0.7259 ± 0.2087 and AUC 0.7962 ± 0.2133.

Based on the scientific studies published in the field which deal with the underlying factors and mechanisms that predispose lymphoma occurrence [9]–[14], we could state that the proposed work constitutes a complementary work with considerable prediction results. Although novel biomarkers have been identified (i.e., BAFF and TNFAIP3 polymorphisms) and validated risk scores have been also developed in terms of clinical parameters [4], [12], [13], [15], we showed that the combination of both data types and the application of ML-based frameworks could result in robust predictive models with impact in the clinical practice. In the era of precision medicine, the exploitation of heterogeneous data types could reveal new knowledge related to the complex molecular mechanisms of cancer development. The relatively small number of SS patients and the class imbalanced problem related to class 1 (i.e., 64 with either a history or a current diagnosis of SS NHL) are the main limitations of the current study. However, given the rates of unrecognized diagnosis of SS patients in the general population as well as the infrequency of SS initial findings in the healthcare sector, the dataset of the present study can be considered as one of the largest SS databases.

## IV. Materials and Methods

### A. Data Collection and Curation

#### 1) Study Cohort

Medical records of 143 primary SS patients (SS) without and 64 SS patients with a history or a current diagnosis of B-cell Non-Hodgkin lymphoma (SS NHL), fulfilling the revised European/American International classification criteria for SS, were collected (Table 1 and supplementary material). DNA derived from whole peripheral blood of 207 patients with primary SS fulfilling the revised European/American classification criteria [28] was collected. The patients were genotyped for 13 single nucleotide polymorphisms (Table 2) after was extracted and stored at −20°C upon use at the Department of Physiology, National and Kapodistrian University of Athens, Athens, Greece. Methods of DNA extraction and genotyping protocols have been previously described in [12], [15]–[19].

**TABLE 1.** The Variables of the Initial Demographic, Clinical and Laboratory Findings Related to the Patients’ Samples Considered in the Current Study. The Mean±SD Values and the Min/Max Values Were Calculated for Continuous Variables. The Respective Percentages Were Also Calculated for the Discrete Variables. These Values Were Computed for Both Classes (I.E. Class 0 = No Lymphoma Development; Class 1 = Lymphoma Development). The Undefined Percentages for Categorical Variables are Also Given

**Category**	**Variable**	**Class 0**			**Class 1**		
		mean	SD	min / max	mean	SD	min / max
Demographic	Age at SS diagnosis (years)	50.93	13.38	15 / 74	50.67	14.27	24 / 81
**Category**	**Variable**	**Class 0 (%)**			**Class 1 (%)**		
		False	True	Undefined	False	True	Undefined
Clinical features	Salivary Grand Enlargement (SGE)	78.32	20.97	0.71	31.25	67.18	1.57
	Raynaud's phenomenon	78.35	21.65	0.00	59.37	40.63	0.00
	Lymphadenopathy	86.00	14.00	0.00	54.68	45.32	0.00
	≥ 40 age at SS diagnosis	82.52	17.48	0.00	25.00	75.00	0.00
Laboratory characteristics	Monoclonal gammopathy	89.51	6.29	4.20	71.87	25.00	3.13
	Anti-Ro/SSA or/and anti-La/SSB positivity	74.12	24.47	1.41	11.00	89.00	0.00
	RF positivity	50.34	41.25	8.41	15.62	82.81	1.57
	Low C4	53.14	45.45	1.41	20.31	76.56	3.13

**Table 2.** The Genetic Variants (Gene Ids and Rs Reference Numbers) Related to the Patients’ Samples Considered in the Current Study. The Percentages for Common Genotype (0), Heterozygous (1), Homozygous (2) and Undefined SNPS Within Both Classes are Presented

**Category**	**GENE/ ID**	**rs #**	**Class 0 (%)**				**Class 1 (%)**			
			0	1	2	Undefined	0	1	2	Undefined
Genetic variant	MTHFR/ 4524	rs1801133	39.80	46.20	14.00	0.00	39.06	42.18	18.76	0.00
	MTHFR/ 4524	rs1801131	55.64	32.16	11.20	0.00	53.12	35.93	10.95	0.00
	TNFRSF13C (BAFF Receptor)/ 115650	rs61756766	93.70	6.30	0.00	0.00	90.60	9.40	0.00	0.00
	TNFSF13B (BAFF)/ 10673	rs1224141	71.32	26.57	1.40	0.71	60.93	35.93	0.00	3.14
	TNFSF13B (BAFF)/ 10673	rs12583006	51.04	35.66	13.30	0.00	53.12	39.06	4.70	3.12
	TNFSF13B (BAFF)/ 10673	rs9514828	15.39	56.64	27.97	0.00	10.93	56.25	29.68	3.14
	TNFSF13B (BAFF)/ 10673	rs1041569	52.44	44.05	3.51	0.00	51.56	39.06	6.25	3.13
	TNFSF13B (BAFF)/ 10673	rs9514827	44.05	44.75	11.20	0.00	35.93	48.43	10.93	4.71
	TREX1/ 11277	rs11797	35.66	44.75	18.90	0.69	34.37	40.62	23.43	1.58
	TREX1/ 11277	rs3135941	69.23	23.07	7.00	0.70	78.12	17.18	3.12	1.58
	TNFAIP3/ 7128	rs2230926	89.51	9.80	0.00	0.69	93.75	6.25	0.00	0.00
	PTPN22/ 26191	rs2476601	89.51	10.49	0.00	0.00	89.06	10.94	0.00	0.00
	LILRA3/11026	deletion	79.00	14.70	0.00	6.30	87.50	6.25	1.56	4.69

Demographic, clinical and laboratory features were recorded after thorough chart review. Lymphoma diagnosis in the SS-lymphoma group was based on the criteria outlined by the World Health Organization classification. This study was carried out in accordance with the recommendations of the Ethics Committee of the National and Kapodistrian University of Athens (approved No. 6337) with written informed consent from all subjects following the Declaration of Helsinki.

#### 2) Data Preprocessing and Curation

Data preprocessing was performed by utilizing an automated framework for evaluating the data quality [29]. The main steps followed towards the dataset quality assessment are referred to the detection of (i) missing values in an autonomous way, (ii) removal of outliers, and (iii) duplicate values and highly correlated distributions among variables. More details are provided within the supplementary material with reference to the preprocessing steps and the data curation procedure that were followed.

### B. Cost-Sensitive Random Forest Feature Selection and Ranking

The RF classifier was applied aiming at evaluating the importance of features with reference to the classification problem (supplementary material). The “balanced mode” of the RF estimator was selected in the current study to automatically adjust weights associated with the class frequencies in the training set. The identification of the most important predictor variables which contribute to accurate and unbiased predictions of the response variable was achieved. The maximum number of features selected after keeping the threshold disabled (i.e., threshold = $ - \infty $) was also reported with reference to the feature ranking results.

### C. Ensemble Methods

Ensemble methods enhance the classification accuracy by aggregating the predictions of multiple base classifiers [32]. During a classification task with ensemble methods a set of base classifiers is developed from the training data and the performance of the classification model is evaluated by voting on the individual predictions made by each classifier. The rationale for ensemble methods is that the error rate during a classifier's performance is considerably lower than the error rate of the base classifiers, considering that the base classifiers are not identical but independent [32].

Let $D$ denote the original training data and $T$ be the test set. A training set ${D_i}$ is created from $D$, which size is kept identical with the original data while the distribution of records may be different. A base learner ${C_i}$ is built from ${D_i}$, for $i = 1, \ldots,k$, which denotes the number of base classifiers. For each test record $x \in T$ to be classified, the predictions made by each base classifier ${C_i}(x)$ are then aggregated by taking a majority vote on the individual base learners predictions in order to obtain the class ${C^*}(x)$:

\begin{equation*}
{C^*}\left(x \right) = Vote\left({{C_1}\left(x \right),\;{C_2}(x} \right),\;.\;.\;.\;,\;{C_k}\left(x \right))\tag{1}
\end{equation*}

Ensemble methods achieve better classification results with unstable classifiers which are sensitive to minor perturbations in the training phase. Examples of such classifiers are the decision trees, the rule-based classifiers and the artificial neural networks [32]. The proposed ML-based methodology enables the minimization of errors related to the variability of the training samples due to the utilization of ensemble algorithms. The bias-variance decomposition method is usually applied for the analysis of such types of errors concerning the predictions of a classification model [32]. In the current study, the GB and RF ensemble classifiers are considered and further implemented [33]–[36] based on imbalanced datasets which consist of categorical variables. We aim to develop predictive models, in terms of machine learning techniques, with high generalization ability and less training errors. More details are given in the supplementary material related to the GB and RF classification models, the performance evaluation and validation along with their parameter tuning.

## V. Conclusions

According to the reported classification results in the current study we could conjecture about the potential of exploiting the clinicogenomic profiles of patients for predicting lymphoma development during SS progression. Based on the proposed ML-based methodology we demonstrated that ensemble methods could obtain promising classification results comparing to conventional statistical methods and/or other supervised learning algorithms used for the development of predictive models in healthcare. Although lymphoma development presents an unmet clinical need in the research field of SS, the international efforts among groups and the conduction of SS prospective studies could provide a clinical impact to the disease management and the patients’ daily activity. Apparently, Genome Wide Association studies (GWAs) could provide observational studies of genome-wide genetic variants which can be easily incorporated in our proposed methodology; thus, enhancing the identification of new population-based risk genetic variants in SS. Towards this direction, the exploitation of large and heterogeneous SS datasets in future multicenter studies could contribute to the development of more accurate predictive models through ML techniques. Furthermore, the rise of omics data and their exploitation in the biomedical sciences could empower the identification of key factors involved in lymphomagenesis and the detection of high-risk patients at early stages.

## Supplementary Materials

In the supplementary material details are given related to the obtained results, the study cohort, the preprocessing steps (i.e. data curation) and the model training and parameter tuning of the ensemble classifiers.


