# Supplementary material for: Predicting Lymphoma Development by Exploiting Genetic Variants and Clinical Findings in a Machine Learning-Based Methodology With Ensemble Classifiers in a Cohort of Sjögren's Syndrome Patients
Source: IEEE Open J Eng Med Biol. 2020 Feb 14;1:49–56. doi: 10.1109/OJEMB.2020.2965191 (PMC8979630; doi:10.1109/OJEMB.2020.2965191)
Supplement: Supplementary file 1 [file supp1-2965191.pdf]

Supplementary Materials

Predicting lymphoma development by exploiting genetic variants and clinical findings in a machine learning-based methodology with ensemble classifiers in a cohort of Sjögren's Syndrome patients

Konstantina D. Kourou, Vasileios C. Pezoulas, Eleni I. Georga, Themis Exarchos, Costas Papaloukas, Michalis Voulgarelis, Andreas Goules, Andrianos Nezos, Athanasios G. Tzioufas, Haralampos M. Moutsopoulos, Clio Mavragani and Dimitrios I. Fotiadis*, *Member, IEEE*

I. Materials and Methods

A. Study Cohort

Demographic and clinical characteristics such as: gender, year of birth, year of disease diagnosis, age at SS diagnosis, ≥ 40 age at SS diagnosis, salivary gland enlargement, Raynaud's phenomenon lymphadenopathy and lymphoma development were recorded. Laboratory data, at the time of SS diagnosis, were collected including monoclonal gammopathy, autoantibodies (antinuclear antibodies, anti-Ro/SSA, anti-La/SSB antibodies, rheumatoid factor [RF], antimitochondrial, and anti-thyroid), cryoglobulins, and C3/C4 protein levels. In the histopathologic lesion of labial minor salivary gland (MSG) tissues, germinal center formation and the presence of monoclonality (as previously described [4]) were also recorded

B. Data preprocessing and curation

The data curation framework enhanced our assessment related to the types of variables included in the raw dataset and their quality in terms of missing and duplicate measurements and outlier's detection. In the case of missing values handling, we excluded subsequently the clinical records that exhibited a percentage of missing values higher than 90%. The variables within the dataset that also exhibited a percentage of missing values higher than 80% were not selected for further analysis. In terms of outliers and duplicate values detection, the respective features were not considered in the cleaned dataset. To complete any missing value detected within the dataset after the data preprocessing and curation step, an imputation transformer was also developed in Python by utilizing the Sci-kit learn library [30]. The "SimpleImputer" transformer was adopted with strategy "mean" for replacing missing values using the mean along the continuous variables, and "most_frequent" for each categorical variable.

After applying the preprocessing procedure, we concluded with 207 patient records consisting of 23 clinical, laboratory, demographic and genetic variables. Table I and Table II present the four different categories of the preprocessed dataset exploited in the current study. The dataset of this study was curated by removing the duplicate features, the patient records with high percentage of missing values and the features detected with outliers. The samples distribution of the categorical features within each class (class 0 = no lymphoma development; class 1 = lymphoma development) was described by the corresponding percentages, whereas for the continuous variables was described by their arithmetic mean and standard deviation values (SD) as well as their min and max values.

C. Cost-sensitive Random Forest Feature Selection and Ranking

The RF classifier was applied aiming at evaluating the importance of features with reference to the classification problem. The "balanced mode" of the RF estimator was selected in the current study to automatically adjust weights associated with the class frequencies in the training set. The identification of the most important predictor variables which contribute to accurate and unbiased predictions of the response variable was achieved. The maximum number of features selected after keeping the threshold disabled (i.e. threshold = $-\infty$) was also reported with reference to the feature ranking results.

According to the binary classification and regression trees and RFs background [31], the best split $s_t = s^*$ at each node t maximizes the decrease:

$$\Delta_i(s,t) = i(t) - P_L i(t_L) - P_R i(t_R), \quad (1)$$

of some impurity measure i_t (e.g. the Gini index, the Shannon entropy or the variance of the response variable Y). t_L and t_R denote the left and right child nodes of node t following split s_t, respectively. The RF algorithm instead of searching for the best split at each node, selects a random subset of K variables and determines subsequently the best split over these features only. To evaluate the importance of a variable X when considering aggregation of randomized trees for predicting response Y, the weighted impurity decrease is used over all N_T trees in the forest [31]:

$$Imp(X_m) = \frac{1}{N_t} \sum_T \sum_{t \in T: v(s_t)=X_m} p(t) \Delta i(s_t, t), \quad (2)$$

where p_t is the proportion $\frac{N_t}{N}$ of training samples reaching node t and $v(s_t)$ is the feature used in split s_t. Equation (1) constitutes the Mean Decrease Impurity (MDI) importance. By sorting the importance scores $imp(X_j)$ in descending order, a ranked list of variables, $R = \left[X_{j'_1,\dots,}X_{j'_d}\right]$ is obtained, where $J' = [j'_1, \dots, j'_d]$, $j'_j \in [1, \dots, d]$ and $imp(X_{j'_j}) \geq imp(X_{j'_{j+1}})$ [31].

D. Model training and parameter tuning with ensemble classifiers

1) Gradient Boosting (GB) classification model

Boosting is a known example of ensemble methods that manipulates the training samples for improving classification accuracy. The overall classification accuracy is obtained by aggregating the predictions of multiple base learners [32]. Boosting methods assign a weight to each training sample and at the end of each boosting round they may adaptively change the weight [33]. GB for classification approximates a simple parameterized function and fits regression tree(s) sequentially on the negative gradient of the specified loss function. Furthermore, GB incorporates randomness into the function estimation procedures for improving the performance. Based on this knowledge, GB constitutes an appealing choice for solving classification problems and building predictive models from an input dataset.

According to (2), the parameters $\{a_m\}_0^M$ and the expansion coefficients $\{\beta_m\}_0^M$ are jointly fit to the training data in a stage-wise manner. Based on an initial guess $F_0(x)$, for each $m = 1,2, \dots, M$ we have:

$$(\beta_m, a_m) = \arg\min_{\beta,a} \sum_{i-1}^{N} L(y_i, F_{m-1}(x_i) + \beta h(x_i; a) \quad (3)$$

and

$$F_m(x) = F_{m-1}(x) + \beta_m h(x; a_m). \quad (4)$$

According to [26], GB solves equation (4) for differentiable loss function $L(y, F(x))$ with a two-step procedure. In the first step, the function $h(x; a)$ is fit to the current "pseudo"-residuals by least squares:

$$a_m = \arg\min_{a,\rho} \sum_{i=1}^{N} [y_{im} - \rho h(x_i; a)]^2, \quad (5)$$

where y_{im} is the current "pseudo" –residuals. Then, given $h(x; a_m)$, the optimal value of the coefficient β_m is determined based on:

$$\beta_m = \arg\min_{\beta} \sum_{i=1}^{N} L(y_i, F_{m-i}(x_i) + \beta h(x_i; a_m)). \quad (6)$$

Gradient tree boosting follows this approach considering that the base learner $h(x; a)$ is an L terminal node regression tree. In the current study, the ensemble model selection procedure reveals the efficiency of the gradient tree boosting classifier, which may well reduce model's variance, noise and bias; thus, minimizing model's training error. The proposed ML-based methodology was implemented in Python by utilizing the Sci-kit learn library and the imbalanced-learn toolbox for classification purposes [30, 34]. Gradient tree boosting algorithm was nested into an EasyEnsemble [34] random under-sampling scheme to handle the class imbalance problem in our dataset. The EasyEnsemble approach exploits the samples from the majority class that have been ignored by under-sampling. We herein selected the combination of EasyEnsemble with the ensemble strategy of gradient boosting for imbalanced data classification. Given a set of minority class samples P, a set of majority class samples N, where $|P| < |N|$, the number of subsets T to sample from N and the number of iterations to train the gradient boosting ensemble s_i, EasyEnsemble method randomly samples a subset N_i, $|N_i| = |P|$ for learning an ensemble of balanced gradient tree boosting classifiers trained on different balanced bootstrap samples [35]. The output of EasyEnsemble is a single ensemble with s_i gradient boosting classifiers ("ensemble of ensembles") which reduces the bias of model performance while improving generalization of the decision boundary.

2) Random Forest (RF) classification models

RF is a class of ensemble methods which encompasses multiple decision trees using random vectors from the original training data [32]. Predictions made by multiple decision trees are combined via majority voting aiming at improving the classification accuracy. RFs constitute a combination of tree learners (predictors), where each tree is generated according to the values of an independent train set of random vectors. Internal estimates of the generalization error of the combined ensemble trees allow the control and monitoring of error, strength and correlation. Towards this direction, out-of-bag methods have been used aiming at improving internal estimates in relation to the classification accuracy [27]. Internal out-of-bag estimates have also applications to the understanding and measurement of variable importance.

For the k_{th} tree, a random vector Θ_κ is generated which is independent of the past random vectors $\Theta_1, \dots, \Theta_{k-1}$, but with the same distribution. The training set and Θ_κ are used to construct the trees resulting into a classifier $h(x, \Theta_k)$, with x being the input vector. According to the definition in [27] a random forest is a classifier consisting of a number of tree-based classifiers $\{h(x, \Theta_\kappa), \kappa = 1, \dots\}$, where $\{\Theta_\kappa\}$ are independent random vectors with the same distribution and each tree in the forest obtains a vote for the most popular class given input x. According to [32], it has been proven that the upper bound for the generalization error of RFs converges to the following expression, when the number of trees is sufficient large:

$$Generalization\ error \leq \frac{\bar{\rho}(1-s^2)}{s^2}, \quad (7)$$

where $\bar{\rho}$ is the average correlation between the trees and s is a quantity that expresses the "strength" of the tree classifiers. The

strength of a classifier implies its average performance, which is computed probabilistically in terms of the classifier's margin [32]:

$$margin, M(X,Y) = P(\widehat{Y_\theta} = Y) - \max_{Z \neq Y} P(\widehat{Y_\theta} = Z), \quad (8)$$

where $\widehat{Y_\theta}$ is the predicted class of X based on the random vector θ that builds the classifier. The higher the margin, the more likely the classifier predicts a new unseen record.

In the present study, the ensemble model selection procedure reveals that RF is also an appealing choice for ensemble-based classification purposes in order to learn imbalanced datasets and achieve better decision boundaries for the predictive model [36]. The RF algorithm was nested into a random under-sampling scheme included in the imbalanced-learn toolbox for handling imbalanced dataset [30, 34]. Specifically, the balanced RF classifier was implemented which randomly under-samples each bootstrap sample to balance it. Each tree of the forest is provided a balanced bootstrap sample resulting in an ensemble of samples including inner balancing samplers.

E. Performance evaluation and validation

To evaluate the classification performance of our proposed methodology six measures including balanced accuracy, which deals with balanced datasets, sensitivity, specificity, positive predictive value, negative predictive value and AUC were used for both GB and RF models. An external stratified 10-fold cross validation was applied with reference to the feature ranking and the boosting classification scheme in each iteration, allowing for the reduction of the models' variance. The stratified 10-fold cross validator [30] returns stratified training folds by preserving the percentage of samples for each class within the dataset. We should note that an inner 5-fold cross validation was also applied in the proposed ML-based methodology for assessing the exhaustive grid search over specified parameter values for each classifier. Using grid search with a nested cross validation for parameter estimation ensures the optimization of model's parameterization.

II. Results

Table III presents the evaluation performance of the GB and RF ensemble classifiers. For the RF classifier both gini and entropy criterion were applied in order to determine the best way to split the samples. The reported classification results of the proposed methodology are high with balanced accuracies of 0.7626, 0.7590 and 0.7780 for RF Gini, RF Entropy and GB estimators, respectively. The respective mean AUC obtained by the classifiers are considerably high implying the accurate model prediction in terms of sensitivity and specificity as presented in Table III (RF Gini classifier: 0.7988, RF Entropy classifier: 0.7995 and GB classifier: 0.8054) and Fig. 1.

We evaluated the models' performances in terms of certain metrics and hyper-parameter optimization criterion (i.e. balanced accuracy). For each prediction model we specified the hyper-parameters to optimize the models' performance. Specifically, for the GB classifier the "learning_rate", the "n_iter_no_change", the "validation_fraction" and the "n_estimators" were set to 0.1, 10, 0.20 and 200, respectively. For the RF models the hyper-parameters selected were the "min_samples_leaf" and the "n_estimators" which were set to 2 and 200, respectively. Default values were used for the rest hyper-paremeters. Tuning the hyper-parameters of the estimators was done with the grid search optimizer available with the sklearn Python library.

Table III

RF Gini, RF Entropy and GB classification results. Outer stratified 10-fold cross validation for evaluating the models' performances and inner 5-fold cross validated grid search for hyper-parameter optimization have been performed for obtaining the classification results. Certain evaluation metrics have been computed regarding the models' performance. The results are presented as mean±2SD. For each input case, balanced accuracy was selected as criterion within the grid search procedure. We highlighted in bold the best results obtained by either input case 1 (clinical and genetic data), input case 2 (clinical data) and input case 3 (genetic data), for each estimator in order to pinpoint the significant findings of the current study.

Classifier	input case #	Hyper-parameter optimization criterion	Balanced Accuracy		Sensitivity		Specificity		Positive Predictive Value		Negative Predictive Value		AUC	
			mean	2SD	mean	2SD	mean	2SD	Mean	2SD	mean	2SD	mean	2SD
RF Gini Ensemble classifier	input case 1	balanced accuracy	**0.7626**	**0.1787**	**0.8000**	**0.3435**	**0.7252**	**0.1702**	**0.5701**	**0.1795**	**0.8974**	**0.1638**	**0.7988**	**0.2186**
	input case 2		0.7626	0.1705	0.800	0.3188	0.7252	0.1577	0.5696	0.1856	0.8964	0.1569	0.8043	0.2019
	input case 3		**0.5350**	**0.1500**	**0.6880**	**0.3045**	**0.3819**	**0.2624**	**0.3352**	**0.1347**	**0.7389**	**0.1587**	**0.4986**	**0.1791**
RF Entropy Ensemble classifier	input case 1	balanced accuracy	**0.7590**	**0.1837**	**0.8000**	**0.3435**	**0.7180**	**0.1989**	**0.5662**	**0.1868**	**0.8960**	**0.1643**	**0.7995**	**0.1917**
	input case 2		07542	0.1907	0.7833	0.3598	0.7252	0.1577	0.5625	0.2006	0.8900	0.1713	0.8107	0.2040
	input case 3		0.5264	0.1823	0.6428	0.2651	0.4100	0.2907	0.3352	0.1722	0.7142	0.1812	0.4987	0.1557
GB Ensemble classifier	input case 1	balanced accuracy	**0.7780**	**0.1514**	**0.8309**	**0.2594**	**0.7252**	**0.2398**	**0.5921**	**0.2095**	**0.9099**	**0.1284**	**0.8054**	**0.1570**
	input case 2		**0.7509**	**0.1701**	**0.7833**	**0.2812**	**0.7185**	**0.1630**	**0.5591**	**0.1859**	**0.8844**	**0.1455**	**0.8215**	**0.1534**
	input case 3		0.5209	0.1792	0.6880	0.3392	0.3538	0.2872	0.3256	0.1441	0.7279	0.2634	0.4618	0.1996
